# Molecular detection of *Leishmania infantum*, filariae and *Wolbachia* spp*.* in dogs from southern Portugal

**DOI:** 10.1186/s13071-016-1452-2

**Published:** 2016-05-10

**Authors:** Carla Maia, Laura Altet, Lorena Serrano, José Manuel Cristóvão, Maria Dolores Tabar, Olga Francino, Luís Cardoso, Lenea Campino, Xavier Roura

**Affiliations:** Global Health and Tropical Medicine, Medical Parasitology Unit, Instituto de Higiene e Medicina Tropical, Universidade Nova de Lisboa, Lisbon, Portugal; Faculty of Veterinary Medicine, Universidade Lusófona de Humanidades e Tecnologias, Lisbon, Portugal; Vetgenomics, Parc de Recerca Universitat Autònoma de Barcelona, Bellaterra, Barcelona, Spain; Hospital Veterinario San Vicente, San Vicente del Raspeig, Alicante Spain; SVGM, Departamento de Ciencia Animal y de los Alimentos, Facultad de Veterinària, Universitat Autònoma de Barcelona, Bellaterra, Barcelona, Spain; Department of Veterinary Sciences, School of Agrarian and Veterinary Sciences, University of Trás-os-Montes e Alto Douro, Vila Real, Portugal; Department of Biomedical Sciences and Medicine, Universidade do Algarve, Faro, Portugal; Hospital Clínic Veterinari, Universitat Autònoma de Barcelona, Bellaterra, Barcelona, Spain

**Keywords:** Dogs, *Dirofilaria immitis*, *Leishmania infantum*, Portugal, *Wolbachia* spp

## Abstract

**Background:**

Leishmaniosis caused by the protozoan *Leishmania infantum* and dirofilariosis caused by the nematodes *Dirofilaria immitis* or *Dirofilaria repens* are vector-borne zoonoses widely present in the Mediterranean basin. In addition, some studies reported that the endosymbiont *Wolbachia* spp. play a role in the biology and pathogenesis of filarial parasites. The aim of this work was to evaluate the frequency of mono- and co-infections by *L. infantum*, filariae and *Wolbachia* spp. and their association with clinical signs in dogs from the south of Portugal. Leishmanial, filarial and *Wolbachia* spp. DNA were evaluated by specific real-time polymerase chain reaction (qPCR) assays in blood samples from 230 dogs.

**Findings:**

One hundred and thirty-nine (60.4 %) dogs were qPCR-positive for *L. infantum* and 26 (11.3 %) for filariae (24 for *D. immitis* only, one *D. immitis* and for *Acanthocheilonema dracunculoides* and another one for *Acanthocheilonema reconditum* only). *Wolbachia* spp. DNA was amplified from 16 (64.0 %) out of the 25 *D. immitis*-positive dogs. Nineteen (8.3 %) dogs were co-infected with *L. infantum* and *D. immitis*, including the one (0.4 %) *A. drancunculoides*-positive animal. In dogs without clinical signs consistent with leishmaniosis and/or dirofilariosis, *L. infantum* prevalence was 69 %, whereas in those dogs with at least one clinical manifestation compatible with any of the two parasitoses prevalence was 42.7 %. *Leishmania* prevalence was significantly higher in apparently healthy mongrels (77.2 %) and pets (76.9 %) than in defined-breed dogs (including crosses; 58.8 %) and in dogs with an aptitude other than pet (i.e. farm, guard, hunting, shepherd or stray), respectively, whereas in those dogs with at least one clinical sign, the detection of *L. infantum* DNA was higher in males (53.3 %) and in those dogs not receiving insect repellents (52.8 %).

**Conclusions:**

The molecular detection of canine vector-borne disease (CVBD) agents, some of which are zoonotic, reinforces the need to implement efficient prophylactic measures, such as insect repellents and macrocyclic lactones (including compliance to administration), in the geographical areas where these agents are distributed, with the view to prevent infection and disease among mammalian hosts including humans.

## Findings

Canine vector-borne diseases (CVBD) constitute a diversified group of illnesses, which are caused by a multitude of pathogens transmitted by arthropod vectors [1]. In addition to their veterinary importance, dogs play a central role in the transmission cycles of some vector-borne agents by acting as reservoirs and sentinels of human infections, thus making the control of CVBD desirable under the One Health umbrella.

Leishmaniosis caused by the protozoan *Leishmania infantum* and heartworm disease and subcutaneous filariosis respectively caused by the nematodes *Dirofilaria immitis* and *Dirofilaria repens* are three vector-borne zoonoses widely present in the Mediterranean basin, with transmission of the first one by phlebotomine sand flies of the genus *Phlebotomus* and of the last two by mosquitoes mainly from the genera *Culex, Aedes* and *Anopheles* [2, 3]. Other less known filarial worms endemic in Europe such as *Acanthocheilonema dracunculoides* and *Acanthocheilonema reconditum* are transmitted mainly by ticks or by fleas and lice, respectively [4].

Endosymbiont bacteria *Wolbachia* spp. (order Rickettsiales) have been found in several filarial species, such as *D. immitis* and *D. repens*, but not in *Achanthocheilonema* spp. [5]. *Wolbachia* organisms seem to play an essential role in the biology of filarial parasites and in the pathogenesis of infections due to these nematodes, potentially increasing the severity of clinical signs [6, 7].

Canine leishmaniosis is a systemic chronic condition whose clinical manifestations often include lymphadenomegaly, cutaneous alterations, weight loss, ocular signs, epistaxis, onychogryphosis and lameness [8]. Canine dirofilariosis is associated with a dry chronic cough, exercise intolerance, dyspnoea, weakness, weight loss, epistaxis, cyanosis and even congestive heart failure [7]. Both parasitoses are endemic in the south of Portugal. *Leishmania* seroprevalence in dogs has ranged from 3.8 % in randomly screened apparently healthy animals from the Algarve [9] to 40.6 % in dogs from the same region that were clinically suspect of leishmaniosis [10]. The detection of *D. immitis* antigen has ranged from 2.4 % in apparently healthy dogs from Lisbon to 17.1 % in dogs from the Algarve with clinical signs compatible with a CVBD [9]. *Wolbachia* spp. DNA has also been detected in *D. immitis* infected dogs from the centre and south of Portugal [11, 12], and *A. reconditum* and *A. drancunculoides* infections have been reported in animals from the same areas [13, 14]. Furthermore, *D. repens* microfilariae were recently detected in one dog from the Algarve region, the southernmost region of continental Portugal [15].

Vector-borne pathogen co-infections may lead to an increased severity of clinical signs as previously shown in dogs from southeastern Spain with leishmaniosis and/or filariosis [16]. On the other hand, a protective role of *Wolbachia* limiting the severity of leishmaniosis was also observed in dogs co-infected with *L. infantum* and *D. immitis* [16]. As the presence of co-infections may lead to a non-characteristic clinical outcome which will further complicate the diagnosis, treatment and prognosis, together with the zoonotic potential of *L. infantum* and *Dirofilaria* spp., the aim of this work was to evaluate the prevalence of mono- and co-infections by *L. infantum*, filariae and *Wolbachia* and their association with clinical signs consistent with leishmaniosis or dirofilariosis in dogs from the south of Portugal.

From May 2011 to February 2014, a total of 230 dogs from veterinary medical centres and animal shelters in southern Portugal randomly selected (i.e. out of any dog present to the veterinary clinic or any dog living in the shelter) were studied (Table 1). The dogs were from the districts of Setúbal (*n* = 68, including 13 dogs from the contiguous districts of Évora and Beja) and Faro (*n* = 162). Domestic dogs were included after informed consent was obtained from the owners. In the case of stray dogs, a written consent for enrolment was obtained from their legal detainer, i.e. the person in charge of the rescue association.

**Table 1:** Molecular prevalence of *L. infantum*, filariae and *Wolbachia* among dogs with no clinical signs compatible with leishmaniosis or dirofilariosis (*n* = 155)

Variable/category	No. (%) of tested dogs	Percentage (n) of positive dogs			
		PCR	PCR	PCR	≥1 agent
		*L. infantum*	filariae	*Wolbachia* spp.	
Gender	154				
Female	84 (54.5)	70.2 (59)	13.1 (11)	6.0 (5)	72.6 (61)
Male	70 (45.5)	68.6 (48)	11.4 (8)	7.1 (5)	70.0 (49)
Age (months)	143				
[1–11]	23 (16.1)	60.9 (14)	4.3 (1)	0.0 (0)	60.9 (14)
[12–83]	82 (57.3)	74.4 (61)	13.4 (11)	6.1 (5)	76.8 (63)
[84–192]	38 (26.6)	60.5 (23)	15.8 (6)	13.2 (5)	63.2 (24)
Breed	147				
Defined (incl. crosses)	68 (46.3)	58.8 (40)^a^	11.8 (8)	5.9 (4)	63.2 (43)
Mongrel	79 (53.7)	77.2 (61)^a^	12.7 (10)	7.6 (6)	77.2 (61)
Aptitude	155				
Pet	108 (69.7)	76.9 (83)^b^	12.0 (13)	5.6 (6)	79.6 (86)^c^
Other^1^	47(30.3)	51.1 (24)^b^	12.8 (6)	8.5 (4)	51.1 (24)^c^
Housing	145				
Indoors and mixed	75 (51.7)	61.3 (46)	9.3 (7)	5.3 (4)	64.0 (48)
Outdoors	70 (48.3)	75.7 (53)	17.1 (12)	8.6 (6)	77.1 (54)
Repellent insecticides^2^	87				
Yes	30 (34.5)	54.4 (31)	8.8 (5)	3.5 (2)	57.9 (33)
No	57 (65.5)	43.3 (13)	10.0 (3)	6.7 (2)	46.7 (14)
Macrocyclic lactones^3^	54				
Yes	13 (24.1)	38.5 (5)	0.0 (0)	0.0 (0)	38.5 (5)
No	41 (75.9)	31.7 (13)	17.1 (7)	14.6 (6)	36.6 (15)
Total	155 (100)	69.0 (107)	12.3 (19*)	6.5 (10)	71.0 (110)

^1^Farm, guard, hunting, shepherd or stray; ^2^Deltamethrin, flumethrin and/or permethrin; ^3^Ivermectin, mimemycin oxime or moxydectin; ^a^ *P* = 0.026; ^b^ *P* = 0.003; ^c^ *P* = 0.001; *Comprising 18 cases of *D. immitis* infection and one case of *A. dracunculoides* and *D. immitis* co-infection

Whenever available, data on gender, breed, age, life style, living conditions, prophylactic use of sand fly and/or mosquito repellents and of macrocyclic lactones, and clinical status, i.e. absence (Table 1) or presence (Table 2) of at least one sign compatible with leishmaniosis or dirofilariosis) were registered for each dog. Clinical signs comprised anorexia, cough, dermatological manifestations, dyspnoea, epistaxis, exercise intolerance, gastrointestinal alterations, lameness, lethargy, lymphadenopathy, muscular atrophy, onychogryphosis, ocular manifestations, pale mucous and weight loss.

**Table 2:** Molecular prevalence of *L. infantum*, filariae and *Wolbachia* in dogs with clinical signs compatible with leishmaniosis and/or dirofilariosis (*n* = 75)

Variable/category	No. (%) of tested dogs	Percentage (n) of positive dogs			
		PCR	PCR	PCR	≥1 agent
		*L. infantum*	filariae	*Wolbachia* spp.	
Gender	75				
Female	32 (42.7)	28.1 (9)^a^	12.5 (4)	9.4 (3)	34.4 (11)
Male	43 (57.3)	53.5 (23)^a^	7.0 (3)	7.0 (3)	55.8 (24)
Age (months)	67				
[1–11]	1 (1.5)	0.0 (0)	0.0 (0)	0.0 (0)	0.0 (0)
[12–83]	33 (49.3)	39.4 (13)	12.1 (4)	12.1 (4)	45.5 (15)
[84–192]	33 (49.3)	42.4 (14)	9.1 (3)	6.1 (2)	45.5 (15)
Breed	73				
Defined (incl. crosses)	40 (54.8)	40.0 (16)	7.5 (3)	7.5 (3)	42.5 (17)
Mongrel	33 (45.2)	48.5 (16)	12.1 (4)	9.1 (3)	54.5 (18)
Aptitude	75				
Pet	37 (49.3)	40.5 (15)	16.2 (6)	13.5 (5)	48.6 (18)
Other^1^	38 (50.7)	44.7 (17)	2.6 (1)	2.6 (1)	44.7 (17)
Housing	41				
Indoors and mixed	18 (43.9)	27.8 (5)	5.6 (1)	5.6 (1)	33.3 (6)
Outdoors	23 (56.1)	47.8 (11)	17.4 (4)	13.0 (3)	52.2 (12)
Repellent insecticides^2^	55				
Yes	19 (34.5)	21.1 (4)^b^	10.5 (2)	5.3 (1)	26.3 (5)
No	36 (65.5)	52.8 (19)^b^	5.6 (2)	5.6 (2)	55.6 (20)
Macrocyclic lactones^3^	37				
Yes	9 (24.3)	0.0 (0)	0.0 (0)	0.0 (0)	0.0 (0)
No	28 (75.7)	25.0 (7)	10.7 (3)	7.1 (2)	32.1 (9)
Total	75 (100)	42.7 (32)	9.3 (7*)	8.0 (6)	46.7 (35)

^1^Farm, guard, hunting or stray; ^2^Deltamethrin or permethrin; ^3^Ivermectin, milbemycin oxime or moxydectin; ^a^ *P* = 0.050; ^b^ *P* = 0.048; *Comprising six dogs positive for *D. immitis* and another one for *A. reconditum*

This study was ethically approved by the boards of the IHMT-UNL and of the Faculty of Veterinary Medicine (ULHT) as complying with the Portuguese legislation for the protection of animals (Decree-Law no. 113/2013).

Whole blood samples were collected by cephalic or jugular venipuncture and spotted onto filter paper. Samples were dried at room temperature and kept at 4 °C until DNA extraction by a commercial kit (Kit Citogene®, Citomed). Four discs of filter paper (4 mm in diameter each) were incubated with lysis buffer (150 μl) and 1.5 μl of proteinase K (20 mg/ml). Further DNA extraction followed the kit manufacturer’s instructions.

All the samples were submitted for real-time PCR (qPCR) for *L. infantum*, filariae (*D. immitis, D. repens, A. dracunculoides* and *A. reconditum*) and *Wolbachia* spp. *Leishmania* quantitative PCR was performed as previously described by Francino et al. [17]. qPCRs for Spirurida (Filariidae, Onchocercidade and Thelaziidae) and *Wolbachia* were carried out in a final volume of 20 μL using SYBR Select (Life Technologies), 0.1 μM of each primer for *Wolbachia* species (described by Tabar et al. [16]) and 0.5 μM of each primer for Spirurida (5′-CGTAATTTTARTWCTTCTTTTTATGATRCTA-3′; 5′-CCAAAYAAACGWTCCTTATCAGTYAA-3′) and 4 μL of DNA (10–100 ng). The thermal cycling profile was 50 °C for 2 min and 95 °C for 10 min followed by 40 cycles at 95 °C for 15 s and 60 °C for 1 min. The eukaryotic 18S RNA Pre-Developed TaqMan Assay Reagent (Life Technologies) was used as internal reference for dog genomic DNA amplification to ensure proper qPCR amplification of each sample and that negative results corresponded to true negative samples rather than to a problem with DNA loading, sample degradation or PCR inhibition. Positive qPCR controls were obtained from clinical samples that had been previously amplified and sequenced to confirm the pathogen and water was used as a PCR-negative control. The *L. infantum* load in blood was quantified according to Delta Ct, namely: low DeltaCt > 15; medium DeltaCt 5-15; high DeltaCt < 5 and very high DeltaCt negative. Spirurida qPCR positive samples were sequenced by the Big-Dye Terminator Cycle Sequencing Ready reaction Kit (Life Technologies) using the same primers. Sequences obtained were compared with GenBank database (www.ncbi.nlm.nih.gov/BLAST).

Percentages of positivity for *L. infantum*, filariae and *Wolbachia* spp. regarding the independent variables and categories were compared by the Chi-square or Fisher’s exact tests. A *P* value ≤ 0.05 was considered as statistically significant. Exact binomial 95 % confidence intervals (CI) were defined for the proportions. Analyses were performed with SPSS® 21 software for Windows and with StatLib.

This study was carried out with a considerable number of dogs (*n* = 230), which were mainly owned and tame ones. On the contrary, previous investigations on canine filarial infections in southern Portugal had either been carried out exclusively with shelter dogs [13, 14] or with not so many (*n* = 157) domestic dogs [12].

One hundred and thirty-nine dogs were qPCR-positive to *L. infantum*. Out of the 97 dogs tested from November to April (*Leishmania* non-transmission period) 72 (74.2 %; CI: 64.3–82.6 %) were positive, whereas from May to October (transmission period) *L. infantum* DNA was detected in 67 (50.4 %; CI: 41.6–59.2 %) of 133 canine blood samples (*P* < 0.001). The significantly higher (*P* < 0.001) *Leishmania* prevalence obtained in the present study, i.e. 69.0 % (CI: 61.1–76.2 %) in dogs with no clinical signs of leishmaniosis and/or dirofilariosis (Table 1) and 42.7 % (CI: 31.3–54.6 %) in dogs with compatible clinical signs (Table 2) compared with the 1.1 % recently obtained in dogs from the south of the country by conventional PCR [18], could be related to the higher sensitivity of the qPCR, which is able to detect less than one parasite per millilitre of blood [17]. This most recent figure of 69.0 % is more in accordance with previously reported molecular prevalence values clearly above 50 % [16]. Nevertheless, it cannot be ruled out that positive blood results were due to transient contamination during the transmission season and may thus represent just exposure to the parasite rather than an established infection.

A negative association was found between *Leishmania* positivity and clinical signs compatible with leishmaniosis and/or dirofilariosis (69.0 % [Table 1] versus 42.7 % [Table 2]; *P* < 0.001). The fact that more dogs considered clinically healthy harboured *Leishmania* parasites in their circulation might be due to a fact most of the enrolled dogs were randomly selected from those attending veterinarian clinics and, therefore, probably receiving more medical care, better nutrition, and for that reason were less prone to develop disease. The prevalence of *L. infantum* (76.9 %; CI: 67.7–84.4 %; *P* = 0.003) and that of infection with at least one pathogen (≥1 agent; 79.6 %; CI: 70.8–86.8 %; *P* = 0.001) in dogs without compatible clinical signs was significantly higher in pets in comparison with dogs with other aptitudes (Table 1). The present results, therefore, disagree with those that revealed a higher prevalence of infection in guard dogs as compared to pets in the Madrid region, Spain [19]. These latter findings might be somewhat due to a different lifestyle of pets (i.e. urban vs. rural) in distinct geographic areas. The higher prevalence of *L. infantum* DNA in clinically healthy mongrel dogs in comparison with those belonging to defined breeds or their crosses (*P* = 0.026) might be related with a certain level of resistance to disease development and progression by the mixed-breed infected dogs [20].

In those dogs showing clinical signs compatible with leishmaniosis and/or dirofilariosis the detection of *L. infantum* DNA in the blood was significantly higher (*P* = 0.050; Table 2) in males (53.5 %; CI: 37.6–68.8 %). Gender predisposition to the infection has been considered by some authors as a non-determinant factor for leishmaniosis [19, 20], while others have reported a higher prevalence in male dogs [21, 22]. On the other hand, the use of long-acting topical insecticides on dogs has been shown to be an effective measure in reducing the prevalence of *Leishmania* infection [23]. Therefore, it is not entirely surprising that the prevalence of *Leishmania* was higher in those dogs not protected against insects (*P* = 0.048; Table 2). In fact, and although the number of effective molecules for prophylaxis against leishmaniosis has increased in the last few years, our results clearly reinforce the idea that there is still a long way to go regarding the prevention and control of *Leishmania* infection.

Filarial infections were detected in 26 (11.3 %; CI: 7.5–16.1 %) blood samples: 24 (10.4 %; CI: 6.8–15.1 %) dogs harboured *D. immitis* only, one (0.4 %; CI: 0.01–2.4 %) dog *A. reconditum* only and another one (0.4 %; CI: 0.01–2.4 %) *A. drancunculoides* in co-infection with *D. immitis*. This corroborates the previous reports on the circulation of these nematodes in the south and central regions of Portugal [9, 12–14]. *D. immitis* prevalence in the present study (10.9 %; CI: 7.2–15.6 %) was lower (although non-significantly; *P* = 0.111) than the overall prevalence of 15.1 % (105/696) recently obtained in three coastal areas of central and southern-central Portugal [14]. Differences can be related to the dynamics of infection over time, which is probably linked to the distribution and abundance of the vectors and to the surveyed canine population. In fact, in the work performed by Alho et al. [14] all the screened animals were sheltered dogs that lived outdoors and had probably not received any prophylactic measure for heartworm infection, thus being more exposed to vectors and to the agents they might transmit. On the other hand, in the present study most of the tested dogs were pets living partially indoors and receiving veterinary health care. Interestingly, and although the difference was not statistically significant (*P* = 0.562), *D. immitis* DNA was not detected in any of the dogs receiving macrocyclic lactones, suggesting the importance of using this prophylactic measure in areas where dirofilariosis is endemic. As the compliance to the use of ectoparasiticides was not evaluated, it cannot be ruled out whether the reason for no differences between the use of insecticides/repellents and the presence of heartworm infection was due to irregular administration of insecticides.

The detection of *Wolbachia* DNA in 16 (64.0 %; CI: 42.5–82.0 %) dogs positive to *D. immitis* was higher than the 52.6 % (20/38; *P* = 0.372) and 30.6 % (15/49; *P* = 0.006) recently obtained in Portuguese [11] and Spanish [16] dogs, respectively. As reported by previous publications, only *D. immitis* and *D. repens* have ever been found infected with *Wolbachia*, but not all the specimens of these filarial parasites transport the endosymbiont bacteria. Taken together, these findings suggest that in endemic areas a combined PCR for *Wolbachia* and *Dirofilaria* should be performed for the diagnosis of heartworm, in order to avoid a high percentage of false-negative *Dirofilaria* results due to the lack of testing for the endosymbiont.

*Wolbachia* DNA was detected in 5 (31.3 %; CI: 11.0–58.7 %) dogs solely infected with *D. immitis* and in 11 (68.8 %; CI: 41.3–89.0 %; *P* = 0.034) co-infected dogs (11 with *L. infantum-D. immitis* and one with *A. dracunculoides-D.immitis*), reinforcing that this endosymbiont does not seem to be present in *Achanthocheilonema* spp. alone [5]. On the other hand, our results contrast with a previous study carried out in dogs with leishmaniosis and/or dirofilariosis from Spain where the prevalence of the endosymbiont was significantly lower in microfilariaemic dogs co-infected with *L. infantum* [16]. Interestingly, in the present study only three out of the 11 *Wolbachia*, *D. immitis* and *L. infantum* co-infected dogs presented clinical signs compatible with leishmaniosis and/or dirofilariosis, which might have been due to the stimulation of a Th1 type protective-immune response triggered by the nematode and the endosymbiont bacterium [16, 24].

In the present study, 19 (8.3 %; CI: 5.0–12.6 %) dogs were co-infected with *L. infantum* and *D. immitis*. It should be stressed that the occurrence of co-infections with these vector-borne agents, which are relatively common in dogs living in geographic areas where competent vectors for the different pathogens co-exist, might induce more severe and atypical clinical outcomes that will further complicate diagnosis, treatment and prognosis [9, 16, 25]. However, this hypothesis has not been corroborated in the present study and the reason might be the random screening of the canine population, with most dogs showing no clinical signs, even when they were positive for one or more of the studied agents.

The on-going and recurrent detection of zoonotic vector-borne agents in dogs with or without clinical signs reinforces the importance of increasing the veterinary community, owners and public health authoritiesʼ awareness regarding the risk of infection. It also highlights the need to apply efficient prophylactic measures, such as insect repellents and macrocyclic lactones (including compliance to administration), not only by the local owners but also by the tourists from non-endemic countries vacationing with their dogs in endemic areas, in order to prevent infection and disease among mammalian hosts including humans. Nevertheless, as environmental changes, global warming, and growing world trade and animal transportation, including the increased mobility of dogs across borders, have an impact on the geographic distribution, abundance and vectorial capacity of arthropods, the expansion of *L. infantum* and filariae to new locations should not be neglected. The present results further stress the need for sustained development of multi-pathogen detection methods in regions endemic and even non-endemic for CVBD.
